# Cognitive, physical and emotional determinants of activities of daily living in nursing home residents—a cross-sectional study within the PROCARE-project

**DOI:** 10.1186/s11556-023-00327-2

**Published:** 2023-09-11

**Authors:** Bettina Wollesen, Nadja Schott, Thomas Klotzbier, Laura Luise Bischoff, Thomas Cordes, Julian Rudisch, Ann-Kathrin Otto, Katharina Zwingmann, Claudia Hildebrand, Thomas Joellenbeck, Lutz Vogt, Daniel Schoene, Matthias Weigelt, Claudia Voelcker-Rehage

**Affiliations:** 1 Department of Human Movement Science, University of Hamburg, Turmweg 2, 20148 Hamburg, Germany; 2 Department of Sports and Movement Science, University of Stuttgart, Stuttgart, Germany; 3 Department of Human Movement Science, University of Vechta, Vechta, Germany; 4 Department of Neuromotor Behavior and Exercise, Institute of Sport and Exercise Sciences, University of Münster, Münster, Germany; 5 Institute of Human Movement Science and Health, Chemnitz University of Technology, Chemnitz, Germany; 6 Institute of Sports and Sports Science, Karlsruhe Institute of Technology, Karlsruhe, Germany; 7 Department of Sport & Health Sciences, University of Paderborn, Paderborn, Germany; 8 Institute of Sports Sciences, Goethe-University Frankfurt, Frankfurt, Germany; 9 Institute of Medical Physics, Friedrich-Alexander University of Erlangen-Nürnberg, Erlangen, Germany

**Keywords:** Cognition, Emotion, Mobility, ADL, Barthel-Index

## Abstract

**Background:**

Interdependencies of health, fitness, cognition, and emotion can promote or inhibit mobility. This study aimed to analyse pathways and interactions between individual subjective and objective physical performance, cognition, and emotions with activities of daily living (ADLs) as mobility indicators in multimorbid nursing home residents.

**Methods:**

The study included *n* = 448 (77.1% females, age = 84.1 ± 7.8 years) nursing home residents. To describe the participant's demographics, frailty, number of falls, and participating institutions' socioeconomic status (SES) were assessed. ADLs were measured with the Barthel Index (BI; dependent variable). Independent variables included objective physical performance, subjective physical performance, cognition, and emotions. A structural equation model (SEM) with maximum likelihood estimation was conducted with AMOS. Direct and indirect effects were estimated using standardized coefficients (significance level of 0.05).

**Results:**

Indices showed (Chi^2^(148) = 217, PCMIN/DF = 1.47; *p* < .001; Comparative Fit Index = .940; Tucker Lewes Index = .902, RMSEA = .033) that the model fitted the data adequately. While there was no direct association between emotions, subjective physical performance, and ADLs, objective physical performance and cognition predicted higher ADLs (p < .01). Emotions had a strong relationship with subjective physical performance, and cognition had a moderate relationship with objective physical performance.

**Discussion and conclusion:**

Objective performance and cognition predicted higher functional status, as expressed by higher BI scores. ADLs, such as mobility, dressing, or handling tasks, require motor and cognitive performance. Subjective performance is an important predictor of ADLs and is only partly explained by objective performance, but to a large extent also by emotions. Therefore, future interventions for nursing home residents should take a holistic approach that focuses not only on promoting objective physical and cognitive performance but also on emotions and perceived physical performance.

**Trial registration:**

Trial registration number: DRKS00014957.

**Supplementary Information:**

The online version contains supplementary material available at 10.1186/s11556-023-00327-2.

## Introduction

Due to demographic change, the number of very old adults that are dependent on professional care has risen sharply. Nursing home residents in long-term care suffer from multimorbidity and an increasing need for complex care interventions. More than half have severe cognitive impairments and/or show signs of dementia [1].

Cognitive decline is often accompanied by functional deficits in strength, balance, walking ability, and complex motor-cognitive tasks, such as dual-tasking [2, 3], limiting the ability to perform (instrumental) activities of daily living (ADLs) independently. This cognitive and physical decline is associated with physical and social inactivity, which may result in additional health-related problems, such as a progression of chronic diseases, depressive symptoms, reduced life-space mobility [4], and reduced quality of life [5].

In addition to factors of physical and cognitive decline that limit ADLs, Webber’s et al. [6] general definition of mobility as well as the new mobility framework for nursing home residents by Vogel et al. [7], assume additional determinants such as social affairs and leisure, as well as internal factors of care facilities and personal factors not only to intercorrelate with mobility but also with ADLs. Besides the abovementioned cognitive and physical functions, personal characteristics such as perceived physical functioning, emotions, and self-efficacy might influence ADLs. Therefore, they might determine the often observed sedentary behavior in nursing home residents, even if they still have the required cognitive and motor resources.

### Subjective and objective physical performance as determinants of ADLs

Older community-dwelling adults merely show reduced muscle strength and physical fitness, often evidenced by decreased grip strength [8], limited functional reach, or the inability to rise from a chair. These objective physical decrements affect mobility and ADLs performance. Specifically, range of motion and balance performance, walking capacity, and walking speed determine ADLs [9]. Moreover, slow walking speed significantly predicts mortality and disability, promoting dependencies in ADLs [3].

After moving to a nursing home, most residents are physically inactive, spending most of their time sitting or lying [10], negatively influencing their physical performance. Furthermore, mobility restrictions resulting in sedentary behavior reduce social interactions and limit cognitive stimulation [11]. Conversely, increasing physical and cognitive performance limitations minimize physical activity and mobility [3]. Therefore, objective physical performance is not only assumed to determine ADL performance but is also associated with other factors, such as subjective physical performance, cognition, and emotions (cf. below).

Concerning subjective performance, subjective beliefs influence performance parameters and might determine the physical activity level. For example, some evidence shows that aspects of self-efficacy are associated with functional limitations and sedentary behavior [12]. In addition, fear of falling in daily life contributes to residents' functional impairment [13], which is thought to impact postural control directly and may cause impairments in ADLs. Moreover, the fear or concerns of falling can lead to an increasingly sedentary lifestyle in community-dwelling older adults [14] and in residents of assisted living homes [15], as well as to solation in nursing home residents [13]. In summary, the effects of objective and subjective physical performance on mobility have already been examined independently. Still, few studies analysed possible interactions or weighted objective and subjective performance in this interaction [16, 17].

### Cognitive performance and emotions as determinants of ADLs

ADLs are also associated with cognitive performance and emotional well-being [18]. Especially executive functions (EF) are necessary to interact with the environment during walking and ADLs. The three main domains of inhibitory control, working memory, and cognitive flexibility are particularly important for ADLs [19]. Typically, these EFs decline with age, accompanied by a reduction in the efficiency of information processing [20]. Cognitive decline exceeding a certain threshold can lead to reduced ADL performance [21]. In addition, cognitive decline often manifests in reduced performance of secondary tasks (dual-tasking), e.g., walking and following signs to navigate the environment [11, 22, 23]. However, the reported studies did not directly examine the interaction of EF, dual-tasking, and ADLs.

Regarding the emotional state in nursing home residents, various aspects can be summarized that influence the emotional state. In addition to specific adaptation to the new environment, social isolation, quality of care, life satisfaction, physical and mental health, depression, and functional impairment also contribute to emotional state. Evidence suggests that various psychological expressions, such as psychological well-being, depression, and life satisfaction, are linked to positive and negative affect [24–26]. Therefore, in this study, we use emotion as an umbrella term including mental well-being and depression, as well as satisfaction with life.

The emotional state also affects ADL performance. There is a high prevalence of depressive symptoms in cognitively unimpaired and impaired nursing home residents [27], which seems to be also associated with a performance decline of ADLs [18]. Depression and changes in emotional states can lead to a reduced sense of well-being and self-esteem [28] and, in turn, reduced activity and ADLs performance. However, findings for nursing home residents are inconsistent regarding the association between the level of physical performance and mental health [16].

Risk factors and symptoms act in concert [17]. The interaction of the different factors (multimorbidity, cognitive and physical decline, reduced mobility, negative emotions) might influence individual trajectories of ADL decrements [17]. Further, exploring this interaction of perceived and objectively measured physical performance and determining its influence on independence and quality of life expressed by ADLs might be an important step toward appropriate and tailored health promotion programs for nursing home residents to counteract inactivity and detrimental health effects. In summary, functional mobility or ADLs, respectively, seems to be determined by a complex interplay of several influencing factors, such as objective and subjective physical performance as well as cognitive performance and emotions. Less is known, however, about how these factors interact in multimorbid residents of long-term care facilities. Therefore, we aimed to analyse them against the background of the additional factors of the mobility framework to gain more insight into their impact on ADLs in nursing home residents.

### Objectives

The main objective of this study was to analyse pathways and interactions between individual objective and subjective physical performance, cognition, and emotions with ADLs. The main research question was: Do objective and subjective physical performance, cognition, and emotions equally predict ADL performance? We hypothesized that within the target group of multimorbid nursing home residents, the emotional status, including depressive symptoms, and the individuals' well-being show the same impact on ADL performance as cognitive objective and subjective motor performance. The potential pathways may help identify interactions that should be targeted in future training interventions to improve ADLs in nursing home residents.

## Materials and methods

### Study design

This cross-sectional study was part of PROCARE, a multicenter study focusing on prevention in long-term care. A detailed study protocol is available at DRKS.de (DRKS00014957; [29]). The Ethics Committee of the Hamburg Chamber of Physicians (registration number PV5762) approved the study. The STROBE Statement [30] guided this study report.

### Participants

The study population consisted of *n* = 448 (77.1% females, age = 84.1 ± 7.8 years) residents of 47 care facilities from eight regions in Germany (cf. [29]). Participants who met the following criteria were included: I) ambulatory, II) able to participate in group activities, III) able to understand and carry out simple instructions, and IV) willing to participate. Nurses suggested participants based on these criteria. All individuals who met the inclusion criteria and their relatives were informed about the study in writing and verbally at each site. They were then asked if they would participate in the study and were given an informed consent form to be signed by the participants or their legal guardians.

### Measures

#### Screening

##### Demographics

Demographics, including age (years), anthropometrics (body height (m), body mass (kg), were collected, and the Body-Mass-Index (BMI) was calculated. The investigators supervising their respective regions judged participating institutions' socioeconomic status (SES). Besides the price category of an institution, its location and surroundings were considered for rating the SES. As a result, the institutions were categorized as low, medium, or high SES.

##### Frailty

Frailty was classified using the phenotyping method [31] based on five items: 1) unintentional weight loss of more than 4.5 kg in the past twelve months (assessed by nursing staff), 2) self-reported exhaustion based on two items of the Center of Epidemiological Studies-Depression Scale (CES-D, see below, 3) weakness (BMI- and sex-adjusted hand grip strength, dominant hand), 4) slowness (height- and sex-adjusted slow walking speed, time to walk a 4.57-m track assessed with a stopwatch), and 5) low physical activity (modified Minnesota Leisure Time Physical Activity Questionnaire; [32]). Each of the five items was rated 0 or 1 and summed up to a total score. Residents were classified as robust (0 points), pre-frail (1–2 points), or frail (3–5 points).

##### Number of falls

The nursing staff documented the number of falls occurring within the last six months.

#### Dependent variable

ADLs were assessed by use of the Barthel Index. The Barthel Index (BI; [33]) is a questionnaire-based measure assessing independence during ADL participation. The BI is recommended for patients and shows good reliability [34]. In each facility, one experienced and trained caregiver evaluated independence in ten categories (e.g., feeding, transferring from bed to chair, personal hygiene, walking, ascending stairs, and dressing). They score on scale levels 0, 5, 10, or 15, depending on the item. Scores range from 0 (entirely dependent) to 100 (fully independent).

#### Independent variables

We used a series of questionnaire-based measures and physical tests to assess participants' performance with high specificity to the target group.

##### Objective physical performance

Hand grip strength was measured using a hand-held hydraulic dynamometer (JAMAR®). Participants were asked to squeeze the dynamometer with maximum isometric effort while sitting on a chair and keeping their arm on an armrest with their elbow bent at an angle of 90° three times (dominant and non-dominant hand). The best result from the dominant hand was used for further analysis. Cut-off values suggested by Fried et al. [31] were used to classify grip strength.

Sitting balance was measured using a modified Functional Reach (FR; [35]) test version. Participants extended their arms forward (shoulders elevated to an angle of 90°) while sitting next to a tape measure attached to the wall and moved their hands forward as far as possible without changing their base of support. The spatial difference in hand position, measured in cm, between upright and total forward movement of one trial was recorded as the participant's score. The normative value for FR is 26.6 cm for community-dwelling older adults [36].

Static and dynamic balance while standing and walking was evaluated with the Short Physical Performance Battery (SPPB; [37]), a reliable and valid assessment for older adults' physical performance, measured balance, and lower extremity functioning (including standing balance measured in closed foot position, semi-tandem and tandem stance, habitual gait speed, chair stand test). In addition, gait speed was assessed as the time required to complete a 4 m walking track. Participants were asked to stand at a line and were instructed to walk down the track at their usual speed as if walking down an aisle in their nursing home. The time it took the participants to complete the 4 m track without deceleration was measured within two trials using a stopwatch or an instrumented gait analysis system (depending on the technical equipment at the different sites). A five-times sit-to-stand transfer was completed for the Chair Stand Test as fast as possible without using the arms. The three tasks were scored from 0 to 4, with higher scores representing better performance.

During all physical measurements, a safe environment with stabilizing equipment (e.g., table, chair, wall) was provided in case of balance loss. For gait assessments, a second test administrator assisted in enabling safety.

##### Cognition

Global cognition was assessed using the Montreal Cognitive Assessment (MoCA; [38]. MoCA scores range from 0 to 30. A score of ≥ 26 reflects cognitive health. Nasreddine et al. [38] report a sensitivity of 90% and a specificity of 87% regarding mild cognitive impairment.

Working memory was assessed using the Serial Subtraction Test (SST). The SST involves counting backward in steps of seven, showing sufficient test–retest reliability (0.80–0.95; [39]. To meet the level of cognitive function present in our study population, we adapted the incremental steps to one and three. Participants counted backward in steps of 1 for 15 s in one trial, starting randomized from 200 or 300. Participants counted backward in steps of 3 in a second trial, starting randomly at 153 or 183. The number of correct answers within one trial period was documented for each trial. Subsequently, correct subtractions after an error occurred were counted as correct answers. The SST was tested in a single-task (sitting) and a dual-task condition (walking), performing the two trials of the SST in a randomized order. Participants were instructed to walk on the track while counting backward until a “stop”-signal was given after 15 s. The trial version and starting number were explained immediately before the “start”-signal. Participants were encouraged to keep on walking during the SST.

##### Subjective physical performance

We used the 12-item *Short-Form Health Survey* (SF-12; [40] for the assessment of quality of life, representing physical (SF-12 phys) and mental health (SF-12 ment—see below) as two components within the independent variable measures. The questionnaire contains twelve items with which participants rate their quality of life. SF-12 physical and mental component summary scales are scored using norm-based methods. The scales' internal consistency (Cronbach’s α) ranges between 0.57 and 0.94 [40].

Concerns about falling were assessed by the short form of the Falls Efficacy Scale-International (Short-FES-I). It is a seven-item questionnaire and provides information on how concerned participants are about falling while executing ADL (e.g., dressing or undressing, showering or bath, getting up from a chair, or sitting down). The scoring of each item of the Short-FES-I ranges between one and four (Cronbach's alpha 0.92, intra-class coefficient 0.83; [41].

##### Measures of the emotional state

The short form of the *CES-D*, an eleven-item questionnaire valid for research on elderly populations [42], was used to screen for depressive symptoms and mood disorders. Answers were given on a scale ranging from “rarely / not at all” (0 points) to “sometimes” (1 point) and “mostly / all the time” (2 points). The scoring of positive items (questions 3 and 11) is reversed. Finally, a sum score from all items was calculated, ranging from 0 to 22.

Life satisfaction was assessed by the *Satisfaction with Life Scale* (SWLS; [43], including global cognitive judgments of satisfaction with one’s life on a seven-point Likert scale from 7 (total agreement) to 1 (no agreement at all). For the SWLS, a total sum was calculated with a possible range of 5 to 35 points. The SWLS has been demonstrated to have strong internal consistency with Cronbach’s alpha of 0.92 [43].

All measurements except for the Barthel Index were conducted by trained research personnel of the included centers of this study according to a standardized research manual.

### Procedures

For rating on a daily routine, inclusion criteria (ambulatory, participation ability, instructions) were estimated by caregivers of the respective institution in consultation with the test supervisors. Measurements followed a standardized protocol. Except for the BI, every measure of one participant was taken by the same test administrator.

### Statistical analysis

IBM SPSS 27.0 (RRID:SCR_016479) and IBM SPSS AMOS 27.0 (RRID:SCR_022686) software was used to perform descriptive analyses and structural equation modeling (SEM).

Some variables in the primary survey contain missing values to varying degrees, and each is related to different aspects of the survey design or the respondents (see Table 2). The proportions of missing data ranges from 2.0% (grip strength) to 34.1% (dual-task). Comparison of missing and non-missing cases by variables of the study can be seen in Table S1 in the Supplementary file. There were some significant differences with, however, small effect sizes for Barthel-Index, Functional Reach, MoCA, and Single & Dual-Task (1er). Little’s test of data Missing Completely at Random (MCAR) was non-significant for MoCA (*p* = 0.507), CES-D (*p* = 0.452), FES-I (*p* = 0.395), SF12 (*p* = 0.056), SWLS (*p* = 0.051). The values of kurtosis and skewness fall within acceptable range of -2 to 2 [44]. Multivariate normality was tested across the variables in the modell using Mardia’s normalized estimate of multivariate kurtosis. In the present study the critical ratio of Mardia’s kurtosis did not exceed the recommended cut-off value of 7 suggesting multivariate normality (see Table S2; [44]). Therefore, strategies for dealing with missing values were adapted in detail to the variables or questionnaires concerned. Sensitivity analyses were performed to examine the influence of missing data. An EM-algorithm was used to estimate missing values in the items of the SF-12 and the CES-D because it reproduces actual patient data most accurately [45]. Missing values of the FES-I and SWLS were handled according to the rules of the scales [41, 43].

t-tests ascertained whether there were differences between males and females for the demographics and (M)ANOVAs with sex as independent variable and age as covariate for the main risk factors measures. Cohen's d was employed to compute effect sizes when comparing the two groups.

A multigroup structural equation modeling (SEM) analysis using SPSS Amos was conducted to examine gender differencesin the hypothesized direct and indirect relationships between BI, objective and subjective physical performance, cognition, and emotion. Direct and indirect effects were estimated using standardized coefficients, adopting a significance level of 0.05. Standardized coefficients with values 0.10–0.29, 0.30–0.49, and > 0.50 were interpreted as small, medium, and large effects, respectively [42]. The Bentler's Comparative Fit Index (CFI), the Goodness of Fit Index (GFI), and the Tucker-Lewis Index (TLI) were used to assess the quality of adjustments of measurement and structural models. These indices signify a good adjustment when values > 0.90 are reached. Root mean squared error of approximation (RMSEA) was also used. A value below 0.10 was considered an indicator of reasonable adjustment. In addition, the absolute index χ2/df was adopted since this indicates an acceptable adjustment for a value < 3 [42].

## Results

### Participants

Table 1 shows the baseline characteristics and demographic data of the included residents. The sample consisted of *n* = 448 participants between 51 and 100 years old (M = 84.1, SD = 7.8; 50–59 = 1.1%, 60–69 = 3.4%, 70–79 = 20.8%, 80–89 = 50.0%, 90–100 = 24.7%). Most of the participants were women (77%). At baseline, this cohort of nursing home residents had a mean (SD) MoCA score of 14.6 (6.62). A cut-off of 22 or lower on the MoCA is used to classify individuals with mild cognitive impairment or dementia, which is the case for 62.6% of our participants. The majority of the participants were normal-weighted, and the prevalence of overweight and obesity was 30% and 26%, respectively. About 50% of the respondents belong to the middle SES group.

**Table 1:** Baseline characteristics of the study population by sex (*n* = 448; means and SD)

	**Men**^**a**^	**Women**	**Statistical analysis**
	*n* = 102	*n* = 343	
**Age (years)** (*n* = 441)	81.7 ± 7.34	84.9 ± 7.79	*t*(439) = -3.69**, d = -0.42
**MoCA (points)** (*n* = 423)	15.6 ± 6.04	14.4 ± 6.76	*t*(171) = 1.67 d = -0.18
**Body Height (m)** (*n* = 402)	1.71 ± 0.08	1.60 ± 0.07	*t*(400) = 11.5**, d = 1.40
**Body mass (kg)** (*n* = 386)	79.3 ± 13.5	67.5 ± 15.9	*t*(384) = 6.20**, d = 0.77
**BMI (kg/m**^**2**^**)** (*n* = 386)	27.3 ± 4.49	26.2 ± 5.87	*t*(177) = 1.81, d = 0.19
**SES (%)**			
**low** (*n* = 116)	23.5	26.8	*CHI*^*2*^(2) = 3.40
**medium** (*n* = 223)	57.8	47.8	
**high** (*n* = 106)	18.6	25.4	
**Frailty**			
**weight loss** (*n* = 365)	0.09 ± 0.28	0.07 ± 0.26	*t*(363) = 0.41, d = 0.05
**fatigue** (*n* = 403)	0.37 ± 0.49	0.36 ± 0.48	*t*(401) = 0.08, d = 0.01
**low PA** (*n* = 396)	0.30 ± 0.46	0.31 ± 0.46	*t*(394) = -0.17, d = -0.02
**gait** (*n* = 377)	0.63 ± 0.49	0.74 ± 0.44	*t*(129) = -1.96, d = -0.25
**weakness** (*n* = 402)	0.72 ± 0.45	0.81 ± 0.39	*t*(137) = -1.71, d = -0.22
**Total** (*n* = 447)	1.75 ± 1.05	1.90 ± 1.08	*t*(443) = -1.16, d = -0.13
**robust** (*n* = 47)	9.8	10.8	*CHI*^*2*^(2) = 0.47
**pre-frail** (*n* = 275)	64.7	61.8	
**frail** (*n* = 123)	25.5	27.6	
**Falls in the last**	0.25 ± 0.49	0.38 ± 0.82	*t*(185) = -0.97, d = -0.17
**6 months** (*n* = 187)			

*MoCA* Montreal cognitive assessment, *BMI* Body-Mass index, *SES* Socio-economic status, *PA* Physical activity

^a^The gender of four participants was not reported

^**^p < .01; * *p* < .05

According to the Fried Frailty phenotype model, 27.6% of older nursing home residents exhibited a frailty status, and 61.8% were in an intermediate stage (pre-frailty). Data for falls were only available from 190 participants, with 75.3% reporting no falls at all, 18,4% reporting one fall, and 6.3% reporting more than one fall.

Table 2 reports descriptive statistics of the complete case analysis at baseline for our variables of interest for this cohort. Cognitive performance was more demanding under the dual-task condition than in the single-task condition. There were 1.3% of residents with CESD scores greater or equal to 16. With mental health (SF12), we found very low variation with age and sex but were comparable with the German norm population from 1994. According to the SWLS, 6.6% of the residents are extremely dissatisfied or dissatisfied, and 51.1% are satisfied or extremely satisfied with their life. The FES-I scale showed that 29.5% expressed serious concerns about falling, but 42.1% reported low concerns about falling. Mean scores for the physical health component of the SF12 are slightly lower than the German norm population from 1994. 13% of the residents could not perform the balance test in SPPB. On the gait test in SPPB, only 21.5% had a walking speed of 0.83 m/s or higher. Roughly half of the residents needed more than one minute or could not perform the chair stand test in SPPB (45.7%). Men had significantly higher mean maximum grip strength than women. Following the European Working Group on Sarcopenia in Older People (EWGSOP) guidelines, 17.2% of the participants can be classified in the sarcopenia group.

**Table 2:** Measures of objective physical performance, cognition, emotion, and subjective physical performance by sex (*n* = 448)

	**Men**	**Women**	**Statistical analysis (F, η**^**2**^_**p**_**)**	
	*n* = 102	*n* = 343	Sex	Age
***Dependent variable***				
**Barthel** (*n* = 402)	76.7 ± 18.6	73.2 ± 19.0	1.39, .003	3.20, .008
***Independent Variables***				
***Objective physical performance***				
**Grip strength dominant hand (kg)** (*n* = 431)	23.3 ± 8.59	13.9 ± 6.28	50.7**, .170	25.2**, .092
**Functional Reach diff (cm)** (*n* = 365)	33.1 ± 13.0	28.3 ± 11.4	4.69*, .019	5.73*, .023
**Gait speed (m/s)**				
**preferred** (*n* = 332)	0.64 ± 0.29	0.60 ± 0.26	0.23, .001	12.3**, .047
**fast** (*n* = 327)	0.80 ± 0.39	0.74 ± 0.33	0.53, .002	8.19**, .032
**SPPB**				
**Chair Stand** (*n* = 396)	1.10 ± 1.12	0.96 ± 1.22	0.07, .001	11.1**, 043
**Balance Score** (*n* = 421)	2.21 ± 1.30	1.77 ± 1.14	1.12, .004	7.69**, .030
**Gait speed of SPPB (m/s)** (*n* = 404)				
**Total** (*n* = 396)	2.24 ± 1.20	2.14 ± 1.07	0.02, 0.001	14.8**, .056
	5.33 ± 2.70	4.66 ± 2.60	0.43, .002	20.3**, .075
***Cognitive performance***				
**MOCA** (*n* = 423)	15.6 ± 6.04	14.4 ± 6.76	0.13, .001	0.10, .001
**ST (number/time)**				
**1er** (*n* = 325)	0.46 ± 0.27	0.44 ± 0.27	0.03, .001	0.04, .001
**3er** (*n* = 322)	0.26 ± 0.21	0.23 ± 0.19	0.21, .001	0.62, .002
**DT (number/time)**				
**1er** (*n* = 290)	0.35 ± 0.29	0.37 ± 0.29	0.87, .003	0.01, .001
**3er** (*n* = 282)	0.21 ± 0.18	0.22 ± 0.20	0.42, .002	0.03, .001
***Subjective performance***				
**FESI** (*n* = 401)	11.2 ± 4.31	11.8 ± 4.73	1.13, .003	0.21, .001
**SF12 physical health status** (*n* = 413)	41.4 ± 10.0	40.4 ± 10.1	1.37, .004	0.66, .002
***Emotion***				
**CESD** (*n* = 379)	6.93 ± 3.92	6.03 ± 3.97	3.16, .009	3.67, .010
**SF12 mental health status** (*n* = 413)	50.9 ± 10.3	51.0 ± 9.90	1.12, .003	0.66, .002
**SWLS** (*n* = 409)	24.6 ± 6.83	24.9 ± 6.29	0.15, .001	4.57*, .013

*SPPB* Short physical performance battery, *MoCA* Montreal Cognitive Assessment, *ST* Single task, *DT* Dual task, *FESI* Falls efficacy scale international, *CESD* Center of Epidemiological Studies-Depression Scale, *SWLS* satisfaction with life scale

^**^p < .01; * *p* < .05

Multivariate analyses of variance (MANOVA) were conducted for the objective and subjective performance, cognition and emotion to test the hypothesis that there were differences between men and women as well as effects for age (see Table 2). There was neither a gender nor an age effect for the Barthel Index. Multivariate ANOVAs with gender as the independent variable, controlled for age, revealed a significant effect for sex (Wilks-Lambda = 0.66, *F*(7,242) = 17.8, *p* < 0.001, η^2^_p_ = 0.135 and age only for objective performance. Overall, men performed better than women in all motor assessments; younger participants performed better than older ones.

### Correlations between the ADLs and the other observed variables

Table 3 shows the correlation matrix for the observed variables. The BI was positively correlated with objective performance measures (grip strength, gait speed, functional reach) and cognition (MoCA, ST, DT). A high number of correct words per second was positively correlated with the MoCA score. Grip strength and functional reach were positively correlated with gait speed. The correlations were consistent with the pre-specified latent variables.

**Table 3:** Correlation matrix for observed variables

	**MoCA**	**ST-1er**	**ST-3er**	**DT-1er**	**DT-3er**	**CES-D**	**SF-12 men**	**SWLS**	**FES-I**	**SF-12 phys**	**Grip strength**	**Gait normal**	**Gait fast**	**Functional Reach**
**Barthel**	.28**	.20**	.16**	.26**	.11	-.07	.09	.03	-.16**	.16**	.26**	.40**	.42**	.22**
**MoCA**	-	.51**	.59**	.52**	.56**	.08	-.04	-.17**	.21**	-.18**	.13**	.03	-.07	.16**
**ST-1er**		-	.67**	.54**	.51**	.02	-.01	-.08	-.00	-.04	.16**	.15*	.08	.13*
**ST-3er**			-	.51**	.71**	.03	-.02	-.12*	.09	-.04	.17**	.09	.04	.08
**DT-1er**				-	.66**	-.04	.06	-.07	-.02	.01	.08	.26**	.23**	.17*
**DT-3er**					-	-.01	.03	-.07	.07	-.03	.06	.11	.08	.06
**CES-D**						-	-.48**	-.42**	.26**	-.28**	.09	-.10	-.15*	-.03
**SF-12 men**							-	.44**	-.23**	.02	.06	.10	.11	.03
**SWLS**								-	-.24**	.31**	-.02	.07	.13*	-.05
**FES-I**									-	-.50**	-.11*	-.28**	-.30**	-.17
**SF-12 phys**										-	.09	.20**	.28**	.11*
**Grip strength**											-	.21**	.14**	.26**
**Gait normal**												-	.85**	.37**
**Gait fast**													-	.31**
**Functional Reach**														-

^**^p < .01; * *p* < .05

### Evaluation of the proposed model

**Fig. 1:**
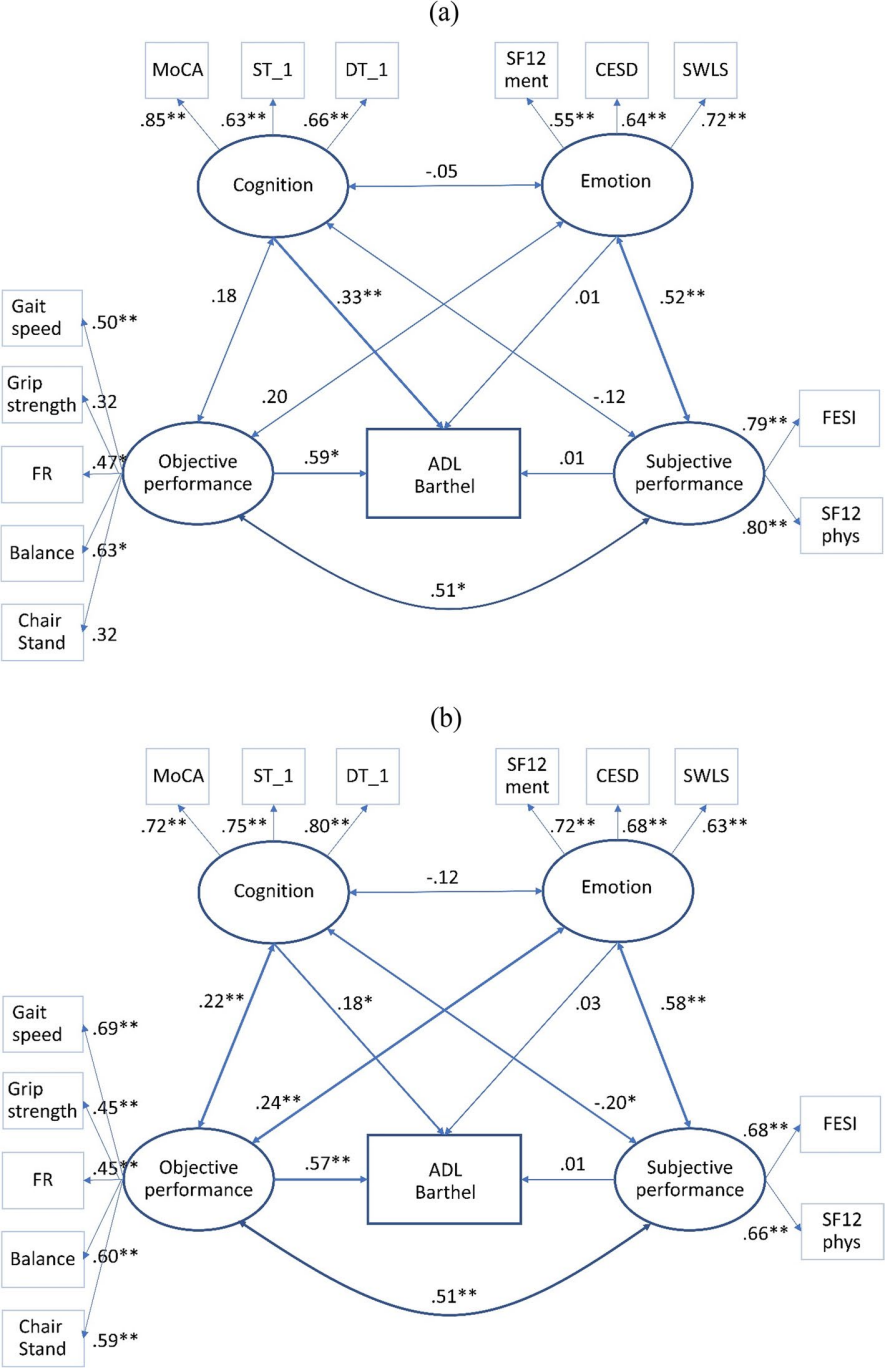
Structural equation model (SEM) of potential predictors of the ADLs (Barthel Index) among older adults living in a nursing home, controlled for gender and age (**a** men, **b** women). Ellipses are used to denote latent constructs, and rectangles are used to denote the observed variables. FR = Functional Reach; MoCA = Montreal Cognitive assessment; ST = Single Task; DT = Dual-Task; SF12 ment = mental health status; CESD = Center for Epidemiological Studies-Depression; SWLS = Satisfaction with Life Scale; FESI = Falls Efficacy Scale International; SF12 phys = physical health status

The model was tested to examine the hypothesized associations between the latent variables for men and women, separately (Fig. 1a and b). Model fit indices demonstrated a good fit CHI^2^(148) = 217, PCMIN/DF = 1.47, *p* < 0.001; Comparative Fit Index = 0.940; Tucker Lewes Index = 0.902, RMSEA = 0.033). While there was no direct association between emotion as well as subjective performance and ADLs (BI), objective physical performance and cognition positively and significantly predicted higher ADLs in men and women. However, emotion had a strong relationship with subjective performance and cognition had a significant relationship with objective physical performance, but only in women. Age was only shown to be a significant variable in perceived performance for men (β = -0.257, *p* = 0.032). For women, age was only significant for objective performance (β = -0.332, *p* < 0.001).

## Discussion

This study aimed to examine pathways and interactions of the individual’s subjective and objective physical performance, cognition, and emotions on ADLs. The SEM analysed objective performance measures as well as subjective outcomes. Overall, the results did not confirm our hypothesis that all analysed dimensions (objective and subjective physical performance, cognition, and emotion) impact ADLs similarly. The main results showed that objective physical performance and cognition positively predicted higher ADLs. However, there was no direct association between subjective physical performance and emotion and ADLs (BI). Moreover, subjective performance was strongly correlated with emotions and objective performance. Results support the importance of cognitive and physical functioning for ADLs in multimorbid nursing home residents.

### Influence of objective physical performance on ADLs

The SEM's factors describing objective physical performance were gait speed, sitting and standing balance, chair rise (as part of the SPPB), grip strength, and functional reach. All these factors are attributes of the latent variable. The indicators' grip strength and functional reach show medium effects. Our cohort showed heterogenous results of the included physical performance measures compared to normative data of other studies (cf. Table 2). Nonetheless, our results confirm other studies that have included subjects with a wide range of physical performance and have consistently found that physical performance is related to the prevalence of physical disability or incidental risks [46]. In addition, the observed differences between men and women as well as decreasing functional capacity are in line with previous research as well as normative data [47–49]. Moreover, several studies have already shown strong predictive validity of indicators of motor performance (gait or chair stand test, [50]; handgrip strength, [51] on decrements in ADLs (and IADLs).

More specifically, all these objective measures have been shown to relate to specific domains of the BI, e.g., climbing stairs, dressing, and eating [9, 50]. For example, slow walking speed is a widely used criterion in geriatric assessment and has become a single estimator of frailty and its consequences [52]. Overall, our results indicate the interplay and importance of gait speed, balance, and chair rise in predicting ADLs and highlight the value of these assessments as diagnostic tools. On the practical side, these results accentuate how important it is for nursing home residents to receive targeted preventive or rehabilitative care to maintain or improve physical performance and, thus, promote ADLs.

### Influence of subjective performance on ADLs

In distinction to current literature, no direct association between subjective performance and functional status detected by the BI could be found in the SEM. Within the SEM, subjective performance was composed of fear or concerns of falling and the score of physical well-being (SF-12). Other studies found weak correlations between functional abilities, e.g. basic or instrumental ADLs and physical health-related quality of life in older adults with cognitive impairment [53]. It has to be noted that the concerns of falling were rated relatively low in our sample [41], and physical health was slightly lower than the norm sample [40]. One explanation for the lower concerns of falling of our nursing home residents might be their accessibility to care supplies. Avoiding falling is part of their living situation and their sedentary lifestyle. Therefore, some items of the FES-I (e.g., going up/down stairs; reaching above your head or on the ground, walking up/down a slope; going out to a social event (e.g., religious service, family gathering, club meeting)) might be irrelevant in their daily life.

### Influence of cognitive function on ADLs

Our results demonstrate that the latent construct cognition was positively related to the functional status (BI) in nursing home residents. The observed variables loading on the latent variable cognition are scores on the MoCA [38] and SST during single- and dual-task situations. This positive relationship also confirms previous studies highlighting a connection between cognitive function and functional status in older adults [46]. All items of the BI require some physical functions related to either fine motor control (e.g., eating or grooming) or balance and mobility (e.g., transfer or climbing stairs). Several studies have shown that gait performance is reduced in individuals with cognitive decline or can even be a predictor of the development of dementia (for review, see [54]).

EF are crucial for lower- and higher-level motor functions and, as such, have relevance for the functional status, which is closely linked to physical performance. In addition, EFs play a significant role in facilitating ADLs [55]. This is crucial in older adults since aging may lead to cognitive decline and affect multiple other structures of sensorimotor control, including sensory (tactile or proprioceptive) organs, passive and active structures of the musculoskeletal system, and cardiovascular and respiratory systems. Due to these changes to different physiological systems, older adults deal with higher amounts of uncertainty in motor control and, thus, require an increase in executive control to, e.g., maintain posture during gait [19]. In addition to basic EF, cognitive decline may affect higher-level cognitive functions necessary for ADLs, such as planning or reasoning, for spatial orientation [11]. In sum, cognitive functioning has a small to moderate impact on the functional status, but not exclusively, via the motor control/objective performance pathway.

### Influence of emotion on ADLs

Interestingly, SEM revealed no direct association between the latent variable „emotion“ (SF-12 mental, CES-D, and SWLS) and BI. Overall, we used three scales, including different emotional aspects. The mental SF-12 scale comprises pain, vitality, and mood, the SWLS deals with life expectancies and quality of life, and the depression scale integrates different symptoms of depression like sleep quality, deficits of attention as well as sadness. Our results contrast with Bürge et al. [18], who revealed that depression is a significant risk factor for ADLs in nursing home residents. Also, a recent study reported that poor self-rated health, poor life satisfaction, and depression are the most substantial risk factors for ADL disabilities [56]. Interestingly, we found no direct association between depressive symptoms and ADLs. Whether this is due to our measures of emotions remains speculative.

### Other intercorrelations

SEM also revealed an association between the latent variables (1) „cognition“ and „objective physical performance“, as well as a relationship between (2) “objective” and “subjective physical performance” and “emotions” for women. (1) As discussed above, studies showed that the coexistence of physical and cognitive impairments is associated with the risk of developing dementia [57]. Montero-Odasso et al. [57] showed that frail participants had a higher prevalence of cognitive impairment than those without frailty and that the combination of slow gait and cognitive impairment posed the highest risk for dementia progression (77% vs. 54%). Moreover, Dodge et al. [58] estimated that cognition accounted for 18% to 36% loss in ADLs and 11% to 29% in IADLs in a community-based sample of older adults. The identified relationship between cognition and “objective physical performance” within our SEM might be one potential pathway for these correlations between cognition and ADLs.

(2) The association between “objective physical performance” and “emotions” might relate to study findings that showed that an increase in depressive symptoms in cognitively unimpaired nursing home residents [27] was associated with a decline in physical performance. Furthermore, as reported above, reduced physical fitness and independence led to increased depressive symptoms in nursing home residents [59], which in turn might affect the intrinsic motivation to be physically active or increase sadness or anger according to their own disabilities. Also, Verghese et al. [60] suggested that the underlying processes of mood, cognition, and fitness should be observed in concert to explain performance in ADLs in older adults living independently. Concerning our SEM, one might suggest that these interdependencies could also be true for nursing home residents in long-term care. However, our findings also showed a reciprocal correlation, which means that the interdependencies obtain in both directions.

In addition to the interaction of emotion and objective physical performance, there was a strong connection between emotional variables and subjective physical performance in women. Previous studies examining community-dwelling older adults found more fall-related concerns and anxiety for women than men [61]. The SEM of our study revealed a strong positive relationship between subjective motor performance and emotions, indicating a more indirect effect of emotions on ADLs. In other populations needing care, studies reported an association between the prevalence of fear of falling and activity restrictions [62] which could lead to reduced ADL abilities in women. Despite the evidence of the relationship between physical performance and cognition, subjective physical performance might be a key to the sedentary behavior of nursing home residents who objectively have physical and cognitive resources. They can perform ADLs, but the emotional state combined with the subjective physical performance could be a more significant barrier for activities than the objective physical and cognitive state. Moreover, the behavior might be influenced by the nursing staff's behavior in the environment [63]. Evidence suggests that caregivers fear the residents' potential falls or pain when performing ADLs leading to fear avoidance behavior and activity restrictions [64]. Maybe this relationship is underestimated in daily practice in nursing home settings.

### Implications for future interventions to promote resources for nursing home residents

This cross-sectional study analysed the pathways and interactions of nursing home residents’ health and physical performance, cognition, and emotion regarding their influence on ADLs. The most significant influence was found in this cohort on objective and subjective physical performance and cognition. Therefore, resources for nursing home residents related to physical performance and cognition should be targeted, for example, through exercise programs. In addition, effective interventions to strengthen health resources, maintain independence in ADLs, and prevent or delay disabilities due to functional decline are highly prioritized in healthcare research [65]. Moreover, for nursing home residents, independent of their frailty status, exercise interventions with resistance, mobility, and balance training [66] have been shown to affect independence and the ability to perform ADLs positively. Considering the SEM results, multicomponent exercise interventions (e.g., a combination of strength, endurance, balance, coordination, and task-specific training on ADLs) combined with cognitive exercises [67] might be a solution. Combined cognitive-motor interventions with dual-task exercise can promote cognitive function in community-dwelling older adults and people with dementia [68] and might simultaneously significantly improve motor and cognitive function. Finally, but especially importantly, more attention must be drawn to the perceived motor performance in nursing home residents.

Considering the investigated associations between depressive symptoms, satisfaction with life, and mental health status with fear of falling shown by SEM, future interventions should also adapt to the environment and target individual functional resources of life-space mobility of nursing home residents. Increasing these individual resources for life-space mobility and other ADLs with targeted exercise interventions, including self-efficacy measures, might also positively affect satisfaction with life and emotional well-being. In addition, social participation significantly influences satisfaction with life [5]. Therefore, exercise interventions should be conducted in groups within the living environment.

The study results might encourage caregivers, clinicians, and policymakers to include tailored exercise interventions for nursing home residents to prevent further decline in functional performance and maintain independence in ADLs.

### Strengths and limitations

The study has several strengths but also some limitations. First, it must be noted that the data collection was standardized in a multicenter study within 47 nursing homes all over Germany, in urban and rural areas with high and low social status, and with a large sample size (*n* = 448). To the best of our knowledge, this is the first study covering various nursing homes with different SES. Moreover, the measurements included allow us to gain a holistic view of functional, cognitive, and emotional health and fitness. However, the nurses selected the participants according to their subjective estimation of the eligibility criteria. This might have led to a selection bias.

Unfortunately, we did not integrate measurements of self-efficacy and motivation for being active or mobile. There is some evidence that maintaining self-efficacy and confidence or optimism is particularly difficult under the conditions of multimorbidity and the need for care. Those in need of care often cannot reduce the unpleasant consequences of their diseases or threatening events, such as the increasing loss of physical or cognitive performance. This can lead to changed beliefs about control on a cognitive and emotional level and may have consequences on a motivational level and, in turn, on mobility [5]. Emotionally, the inability to act leads to anger or sadness, up to the intensification of depressive symptoms. Both aspects then contribute to the fact that motivation to be physically active is reduced. Due to the cross-sectional character of this analysis, these relationships need to be confirmed in future longitudinal studies.

Of course, the participants of this study were a vulnerable group, often with a multimorbid status. Moreover, the examined population integrates not an equal number of men and women. This leads to difficulties in controlling for all possible confounders and could lead to substantial heterogeneity within some subscales of the integrated measures. However, gender-related differences were only observed for the functional performance measures. Nevertheless, the overall prediction of factors affecting ADLs in this study cohort can be considered good regarding statistical data from the model fit with comparable effect sizes for both genders.

## Conclusions

Due to demographic change, the number of very old adults dependent on professional care has risen sharply. In long-term care, nursing home residents (NHRs) suffer from multimorbidity and an increasing need for complex care interventions. Cognitive and physical declines are associated with physical and social inactivity, which may result in additional health-related problems, such as a progression of chronic diseases, depressive symptoms, reduced life-space mobility, and reduced quality of life. We explored the fundamental link between mechanisms of aging and analysed pathways and interactions between individual objective and subjective physical performance, cognition, and emotions with Activities-of-Daily-Living (ADL). This cohort's most substantial influence was on objective and subjective physical performance and cognition. Therefore, resources for NHRs related to physical performance, including subjective performance and cognition, should be targeted, for example, through exercise programs. Independent of NHRs' frailty status, exercise interventions with resistance, mobility, and balance training have been shown to affect independence and the ability to perform ADLs positively. Increasing these individual resources, including specific aspects of subjective performance (e.g., raising self-efficacy or reducing fear of falling) with targeted exercise interventions, might also positively affect satisfaction with life and emotional well-being. The results might encourage caregivers, clinicians, and policymakers to include tailored exercise interventions for NHRs to prevent further decline in functional performance and maintain independence in ADLs.

### Supplementary Information

**Additional file 1.**

**Additional file 2: Table S1.** Comparison of missing and non-missing cases by variables of the study. **Table S2.** Descriptive statistics for variables in the model, including skewness and kurtosis.

## Data Availability

All relevant data are within the study, and raw data are available on request by the corresponding author.
