# Supplementary material for: Cognitive, physical and emotional determinants of activities of daily living in nursing home residents—a cross-sectional study within the PROCARE-project
Source: Eur Rev Aging Phys Act. 2023 Sep 11;20:17. doi: 10.1186/s11556-023-00327-2 (PMC10494417; doi:10.1186/s11556-023-00327-2)
Supplement: Supplementary file 1 — Additional file 1. [file 11556_2023_327_MOESM1_ESM.docx]

**SUPPLEMENTARY MATERIAL**

| **Descriptive statistics** | | | | | | | | | |
| --- | --- | --- | --- | --- | --- | --- | --- | --- | --- |
|  | **N** | **Minimum** | **Maximum** | **Mean** | **SD** | **Skewness** | | **Kurtosis** | |
|  |  |  |  |  |  |  | **SD error** |  | **SD error** |
| **Barthelindex** | 406 | 15,00 | 100,00 | 73,9901 | 18,93041 | -,677 | ,121 | -,263 | ,242 |
| **Grip strength dominant hand** | 440 | ,00 | 45,00 | 16,0813 | 7,87281 | ,997 | ,116 | 1,389 | ,232 |
| **Functional Reach diff (cm)** | 368 | -15,00 | 70,50 | 29,4736 | 11,96445 | ,000 | ,127 | ,367 | ,254 |
| **Gait speed (m/s) preferred** | 399 | ,14 | 1,86 | ,6136 | ,26664 | ,773 | ,134 | 1,094 | ,266 |
| **Gait speed (m/s) fast** | 328 | ,11 | 2,42 | ,7528 | ,34700 | ,798 | ,135 | 1,173 | ,268 |
| **SPPB Chair Stand** | 403 | ,00 | 4,00 | 1,0074 | 1,21247 | 1,139 | ,122 | ,317 | ,243 |
| **SPPB Balance Score** | 424 | ,00 | 4,00 | 1,8679 | 1,18960 | ,181 | ,119 | -,839 | ,237 |
| **SPPB Gait Speed** | 407 | ,00 | 4,00 | 2,1622 | 1,09555 | ,398 | ,121 | -1,072 | ,241 |
| **SPPB_total** | 407 | ,00 | 12,00 | 4,7880 | 2,63679 | ,582 | ,117 | -,289 | ,234 |
| **MoCA** | 423 | ,00 | 29,00 | 14,6430 | 6,61699 | -,176 | ,119 | -,679 | ,237 |
| **ST (number/time) 1er** | 328 | ,00 | 1,33 | ,4450 | ,26993 | ,184 | ,135 | ,220 | ,268 |
| **ST (number/time) 3er** | 325 | ,00 | ,93 | ,2357 | ,19287 | ,853 | ,135 | ,644 | ,270 |
| **DT (number/time) 1er** | 292 | ,00 | 1,56 | ,3682 | ,29101 | ,672 | ,143 | ,913 | ,284 |
| **DT (number/time) 3er** | 284 | ,00 | 1,13 | ,2164 | ,19458 | 1,128 | ,145 | 1,686 | ,288 |
