# Supplementary material for: Cognitive, physical and emotional determinants of activities of daily living in nursing home residents—a cross-sectional study within the PROCARE-project
Source: Eur Rev Aging Phys Act. 2023 Sep 11;20:17. doi: 10.1186/s11556-023-00327-2 (PMC10494417; doi:10.1186/s11556-023-00327-2)
Supplement: Supplementary file 2 — Additional file 2: Table S1. Comparison of missing and non-missing cases by variables of the study. Table S2. Descriptive statistics for variables in the model, including skewness and kurtosis. [file 11556_2023_327_MOESM2_ESM.docx]

**Supplementary material**

**Table S1.** Comparison of missing and non-missing cases by variables of the study.

|  | ***Percentage / Mean*** | | |  | | |
| --- | --- | --- | --- | --- | --- | --- |
|  | ***Missing*** | | ***Non-Missing*** | ***Pearson χ2*** | ***t-value*** | ***p*** |
| ***Demographics*** | | | | | | |
| **Age (years)** | 84.2 ± 7.94 | 84.1 ± 7.70 | |  | -0.14 | n.s. |
| **Gender male female** | 22.1 77.9 | 23.8 76.2 | | 0.19 |  | n.s. |
| **BMI (kg/m^2^)** | 26.3 ± 5.42 | 26.6 ± 5.78 | |  | -0.52 | n.s. |
| **SES (%) low  medium  high** | 17.5 58.1 24.4 | 35.0 42.1 22.9 | | 19.1 |  | < .001 |
| **Frailty robust  pre-frail  frail** | 12.1 63.2 24.7 | 8.9 60.3 30.8 | | 2.78 |  | n.s. |
| **Falls in the last  6 months** | 0.35 ± 0.69 | 0.36 ± 0.80 | |  | -0.11 | n.s. |
| ***Dependent variable*** | | | | | | |
| **Barthel** | 71.9 ± 20.5 | | 76.3 ± 17.1 |  | -2.56 | .011 |
| ***Objective physical performance*** | | | | | | |
| **Grip strength dominant hand (kg)** | 15.8 ± 7.66 | | 16.4 ± 8.11 |  | -0.83 | n.s. |
| **Functional Reach diff (cm)** | 27.8 ± 11.5 | | 30.8 ± 12.2 |  | -2.35 | .019 |
| **Gait speed (m/s) preferred  fast** | 0.61 ± 0.27 0.73 ± 0.36 | | 0.62 ± 0.27 0.77 ± 0.34 |  | -0.21 -0.86 | n.s. n.s. |
| **SPPB Chair Stand  Balance Score  Gait speed of SPPB (m/s) Total** | 0.95 ± 1.89 1.76 ± 1.25 2.18 ± 1.09 4.42 ± 2.69 | | 1.06 ± 1.23 1.97 ± 1.12 2.15 ± 1.10 5.17 ± 2.54 |  | -0.94 -1.82 0.32 -2.99 | n.s. n.s. n.s. .003 |
| ***Cognitive performance*** | | | | | | |
| **MOCA** | 13.1 ± 6.70 | | 16.2 ± 6.16 |  | -5.01 | < .001 |
| **ST (number/time) 1er  3er** | 0.40 ± 0.28 0.21 ± 0.19 | | 0.47 ± 0.26 0.25 ± 0.19 |  | -2.18 1.78 | .030 n.s. |
| **DT (number/time) 1er  3er** | 0.31 ± 0.26 0.19 ± 0.18 | | 0.39 ± 0.30 0.23 ± 0.20 |  | -2.08 -1.54 | .038 n.s. |
|  |  | |  |  |  |  |
|  |  | |  |  |  |  |
| ***Subjective performance*** | | | | | | |
| **FESI** | 12.0 ± 4.70 | | 11.4 ± 4.56 |  | 1.34 | n.s. |
| **SF12 physical health status** | 40.4 ± 9.99 | | 40.9 ± 10.2 |  | -0.54 | n.s. |
| ***Emotion*** | | | | | | |
| **CESD** | 6.35 ± 3.85 | | 6.16 ± 4.11 |  | 0.45 | n.s. |
| **SF12 mental health status** | 50.5 ± 10.1 | | 51.3 ± 9.95 |  | -0.87 | n.s. |
| **SWLS** | 25.3 ± 6.23 | | 24.3 ± 6.53 |  | 1.60 | n.s. |

Note. ** *p* < .01; * *p* < .05. SPPB: Short physical performance battery; MoCA: Montreal Cognitive Assessment; ST: single task; DT:dual task; FESI: Falls efficacy scale international; CESD: Center of Epidemiological Studies-Depression Scale,SWLS: satisfaction with life scale

**Table S2.** Descriptive statistics for variables in the model, including skewness and kurtosis.

|  |  | | | | | ***Skewness*** | | | ***Kurtosis*** | |
| --- | --- | --- | --- | --- | --- | --- | --- | --- | --- | --- |
|  | ***min*** | ***max*** | ***mean*** | ***SD*** | ***statistic*** | | ***c.r.*** | ***statistic*** | | ***c.r.*** |
| ***Demographics*** | | | | | | | | | | |
| **Age (years)** | 51.0 | 100 | 84.2 | 7.63 | -0.89 | | -7.62 | 1.57 | | 6.72 |
| ***Dependent variable*** | | | | | | | | | | |
| **Barthel** | 15.0 | 100 | 73.7 | 18.7 | -0.63 | | -5.42 | -0.31 | | -1.34 |
| ***Objective physical performance*** | | | | | | | | | | |
| **Grip strength dominant hand (kg)** | 0.00 | 45.0 | 16.1 | 7.89 | 0.98 | | 8.38 | 1.32 | | 5.64 |
| **Functional Reach diff (cm)** | 0.00 | 66.0 | 29.6 | 11.7 | 0.24 | | 0.21 | -0.19 | | -0.83 |
| **Gait speed (m/s) preferred** | 0 | 1.68 | 0.62 | 0.27 | 0.49 | | 4.22 | 0.27 | | 1.13 |
| **SPPB Chair Stand  Balance Score** | 0 0 | 4 4 | 1.00 1.87 | 1.20 1.20 | 1.14 0.17 | | 9.72 1.49 | 0.32 -0.86 | | 1.39 -3.67 |
| ***Cognitive performance*** | | | | | | | | | | |
| **MOCA** | 0.00 | 29.0 | 14.7 | 6.66 | -0.18 | | -1.51 | -0.68 | | -2.91 |
| **ST number/time) 1er** | 0.00 | 1.33 | 0.44 | 0.26 | 0.23 | | 1.92 | 0.24 | | 1.01 |
| **DT (number/time) 1er** | 0.00 | 1.56 | 0.35 | 0.28 | 0.59 | | 5.08 | 0.45 | | 1.93 |
| ***Subjective performance*** | | | | | | | | | | |
| **FESI** | 7.00 | 28.0 | 11.4 | 4.60 | 1.07 | | 9.12 | 0.76 | | 3.26 |
| **SF12 physical health status** | 13.1 | 63.4 | 40.9 | 10.1 | -0.25 | | -2.17 | -0.64 | | -2.74 |
| ***Emotion*** | | | | | | | | | | |
| **CESD** | 0.00 | 18.0 | 6.17 | 4.00 | 0.47 | | 4.06 | -0.22 | | -0.92 |
| **SF12 mental health status** | 17.9 | 68.9 | 68.9 | 50.6 | -0.77 | | -6.57 | -0.02 | | -0.07 |
| **SWLS** | 5.00 | 35.0 | 24.8 | 6.26 | -0.43 | | -3.71 | -0.51 | | -2.19 |
| **Multivariate** |  |  |  |  |  | |  | 13.7 | | 6.36 |

Note. SPPB: Short physical performance battery; MoCA: Montreal Cognitive Assessment; ST: single task; DT:dual task; FESI: Falls efficacy scale international; CESD: Center of Epidemiological Studies-Depression Scale,SWLS: satisfaction with life scale
